# Small Molecule Modulation of NOS1AP for Molecularly Targeted Glioblastoma Therapy

**DOI:** 10.3390/life16091519

**Published:** 2026-09-11

**Authors:** Ashraf N. Abdo, Sungwoo Cho, Moustafa Gabr

**Affiliations:** 1 Department of Radiology, Columbia University Irving Medical Center, New York, NY 10032, USA; aa6194@cumc.columbia.edu (A.N.A.); sc6090@cumc.columbia.edu (S.C.); 2 Department of Radiology, Molecular Imaging Innovations Institute (MI3), Weill Cornell Medicine, New York, NY 10065, USA

**Keywords:** glioblastoma, NOS1AP, drug discovery, molecular therapeutics

## Abstract

NOS1AP (CAPON) is a signaling adaptor implicated in glioblastoma (GBM) proliferation, but its chemical tractability remains largely unexplored. Here, affinity selection–mass spectrometry screening of 10,000 drug-like compounds identified **NGM1**, a small molecule scaffold that directly binds NOS1AP. Binding was confirmed by microscale thermophoresis (MST), yielding a dissociation constant of 11.9 ± 7.0 μM. Focused structure–activity analysis and molecular modeling identified structural features associated with NOS1AP recognition and provided a framework for compound optimization. In living cells, **NGM1** produced a concentration-dependent reduction in the NanoBRET signal generated by the NOS1AP-NOS1 reporter pair, consistent with perturbation of the complex. **NGM1** reduced viability in U87 and U251 GBM cells, with IC_50_ values of 12.7 ± 0.91 and 17.3 ± 1.04 μM, respectively, while normal human astrocyte viability remained above 50% at 150 μM. Mechanistic studies in U87 cells demonstrated reduced DNA synthesis, G_0_/G_1_ cell-cycle accumulation, and induction of apoptosis, accompanied by increased p53 and CDKN1A and decreased CDK6. Collectively, these findings establish the initial chemical tractability of NOS1AP and identify **NGM1** as a chemical scaffold for investigating NOS1AP-associated signaling in GBM. The findings also provide a foundation for optimizing NOS1AP-directed compounds.

## Simple Summary

Glioblastoma is an aggressive brain cancer for which new therapeutic targets and drug-delivery strategies are urgently needed. NOS1AP is a signaling protein associated with glioma-cell growth, but it has not previously been directly targeted for glioblastoma therapy. This study identifies an initial NOS1AP-binding compound and shows that it can alter a NOS1AP-containing protein complex in living cells. Experiments in two glioblastoma cell models demonstrated concentration-dependent reductions in viability, while normal human astrocytes were substantially less sensitive. The findings provide a foundation for optimizing NOS1AP-directed compounds and developing delivery systems to enhance brain penetration, tumor-selective accumulation, and therapeutic exposure in future studies.

## 1. Introduction

Glioblastoma (GBM) is the most aggressive primary malignant tumor of the adult central nervous system, characterized by rapid proliferation, extensive vascularization, and near-universal recurrence despite aggressive multimodal therapy. Maximal safe surgical resection followed by concurrent chemoradiotherapy with temozolomide, per the Stupp protocol, remains the clinical backbone of first-line management [1,2,3,4,5]. Despite advances in multimodal treatment, contemporary population-level outcomes remain poor. A recent U.S. SEER analysis of 27,534 patients with GBM who underwent surgery and postoperative radiotherapy reported a median overall survival of 15 months for patients diagnosed between 2016 and 2020, compared with 14 months for those diagnosed between 2005 and 2015, indicating only limited improvement in survival during the contemporary treatment era [1].

Several biological features underlie this therapeutic recalcitrance. Diffuse infiltrative growth along white matter tracts precludes complete surgical clearance and allows residual tumor cells to persist beyond resection margins. Marked intratumoral and intertumoral heterogeneity, driven by clonal evolution and the coexistence of distinct transcriptional subtypes (proneural, classical, mesenchymal) within a single tumor, undermines therapies designed against a single dominant molecular driver. Adaptive signaling rewiring, including compensatory activation of parallel receptor tyrosine kinase and downstream effector pathways, allows tumor cells to circumvent pathway-selective inhibition. Compounding these features, an immunosuppressive tumor microenvironment enriched in tumor-associated macrophages/microglia, regulatory T cells, and myeloid-derived suppressor cells limits the efficacy of immune-based interventions. Together, these mechanisms contribute to acquired and intrinsic treatment resistance, frequent local recurrence, and poor long-term survival [6,7,8,9,10,11,12,13].

These persistent limitations have intensified interest in signaling dependencies that fall outside the scope of conventional kinase- and receptor-centered drug discovery programs, including non-canonical scaffolding interactions, epigenetic regulators, and microenvironment-directed targets, as a means of addressing resistance mechanisms that classical approaches have failed to overcome [14,15].

NOS1AP (also known as CAPON) is a multidomain adaptor protein originally identified through its association with neuronal nitric oxide synthase (NOS1/nNOS), where it competes with PSD95 for a shared binding site on nNOS and thereby redirects nNOS signaling away from canonical NMDA receptor-coupled pathways. Rather than catalyzing a biochemical reaction itself, NOS1AP functions as a scaffold, nucleating protein complexes that shape nitric oxide output and downstream signaling cascades governing cell survival, stress responses, and differentiation [16,17,18,19,20]. The gene gives rise to multiple isoforms through alternative splicing, most notably the long form NOS1AP-L, and the biological consequences of NOS1AP expression are strongly context-dependent, varying with cell type, isoform, and the composition of the local signaling complex. This scaffolding function appears to be co-opted in tumor biology: NOS1AP-L overexpression has been reported to exert cell-context-dependent effects in glioblastoma models, inhibiting proliferation in U251 cells while promoting proliferation in U87 cells, with these effects associated with AKT/mTOR/p53 signaling [21]. This finding points to a potential tumor-supportive role for the long isoform, but a genetic or expression-level association alone does not establish whether the underlying scaffolding interaction is druggable or whether pharmacological modulation would translate into a therapeutic effect. Notably, the AKT/mTOR/p53 axis implicated in CAPON-L-driven proliferation is itself a convergence point for multiple oncogenic signaling inputs in glioblastoma, raising the possibility that NOS1AP acts not as an isolated driver but as a modulatory node within pathways already central to GBM pathobiology, a distinction relevant to how selectively a NOS1AP-directed agent might need to act [21]. The work presented here provides an initial chemical entry point for NOS1AP-directed GBM research while defining the validation and delivery challenges that remain for therapeutic development.

Adaptor proteins present a distinct chemical challenge. Their functional surfaces are commonly broad and shallow, they lack catalytic pockets, and structural information may be incomplete. Consequently, conventional activity-based screening can miss ligands that bind outside known interfaces or exert partial allosteric effects. Affinity selection–mass spectrometry (AS-MS) offers a complementary solution by detecting protein-associated small molecules directly in solution without requiring an enzymatic readout or a predefined binding site [22,23,24,25,26,27,28,29,30]. When paired with orthogonal biophysical measurements and live-cell protein-interaction assays, this approach can establish the initial tractability of otherwise understudied signaling proteins.

In this study, we screened a chemically diverse collection of 10,000 drug-like molecules against recombinant full-length NOS1AP-L and identified a NOS1AP-binding chemical scaffold. The lead compound was evaluated through microscale thermophoresis (MST), analog-based structure–activity analysis, pocket prediction, molecular docking, and a live-cell NanoBRET assay measuring the NOS1AP-NOS1 interaction. We then examined whether pharmacological modulation of NOS1AP was associated with antiproliferative activity in two human GBM cell lines and assessed tumor-cell selectivity using nonmalignant human astrocytes. The work provides an initial chemical entry point for NOS1AP-directed GBM research while defining the validation and delivery challenges that remain for therapeutic development.

## 2. Materials and Methods

### 2.1. Protein and Chemical Library

His-tagged recombinant full-length human NOS1AP-L was obtained from Sino Biological. A 10,000-member subset of the Maybridge HitFinder collection was used for primary screening. This diversity-oriented collection was derived from the larger Maybridge Screening Collection using Daylight fingerprint clustering and a Tanimoto similarity threshold of 0.7. Members satisfy conventional drug-like property filters, including molecular weight ≤ 500 Da, calculated logP ≤ 5, no more than five hydrogen-bond donors, and no more than ten hydrogen-bond acceptors, with reported purity of at least 90%.

### 2.2. Affinity Selection–Mass Spectrometry

AS-MS screening was performed with the Automated Ligand Identification System on an Agilent two-dimensional HPLC platform coupled to an Agilent time-of-flight mass spectrometer. His-tagged recombinant full-length human NOS1AP-L (0.1 mM) was prepared in PBS containing 300 mM NaCl and 0.01% Tween-20 in PBS containing 300 mM NaCl and 0.01% Tween-20. Size-exclusion chromatography was driven by an Agilent 1260 HPLC pump and reversed-phase separation was operated by an Agilent 1290 UHPLC pump; the two dimensions were connected through a high-pressure switching valve to an Agilent 6230B TOF mass spectrometer. Positive-ion spectra were processed using Agilent MassHunter and proprietary analysis software. Size-exclusion separation used 700 mM ammonium acetate as buffer A and 70% acetonitrile as buffer B on a Polyhydroxyethyl A column (50 × 2.1 mm, 3 µm, 200 Å; PolyLC). Reversed-phase separation used water containing 0.1% formic acid as buffer A and 90% acetonitrile containing 0.1% formic acid as buffer B on a Kinetex C18 column (50 × 2.1 mm, 2.6 µm, 100 Å; Phenomenex). AS-MS screening was performed using the Automated Ligand Identification System on an Agilent 1260 Infinity HPLC platform coupled to an Agilent 1290 Infinity UHPLC system and an Agilent 6230B time-of-flight mass spectrometer. Mass spectra were acquired and processed using Agilent MassHunter software and proprietary AS-MS analysis software.

The 10,000 compounds were prepared as 10 mM stocks in DMSO and screened as 100 pooled mixtures, each containing 100 compounds. Each compound was diluted to a final concentration of 10 µM in assay buffer during incubation with recombinant NOS1AP-L. A parallel LC-MS run verified compound detectability before affinity selection. Following separation of protein-associated and unbound compounds, recovered ligands were analyzed by LC-MS. Processing of the screening campaign identified 121 initial NOS1AP-associated candidates for follow-up evaluation.

### 2.3. Single-Concentration Biophysical Triage

Fifty candidates selected from the AS-MS campaign were evaluated at a final concentration of 250 µM using a Dianthus instrument (NanoTemper Technologies). Fluorescently labeled His-tagged NOS1AP was combined with each compound in PBST containing 2.5% DMSO. Measurements were performed in triplicate, and changes in normalized fluorescence were compared with the reference signal from PBST containing 2.5% DMSO. Potential hits were defined as compounds with signals outside the mean reference signal ± 3 SD. NGM1 generated a reproducible binding-associated signal in this single-concentration triage assay and was prioritized for further characterization (Figure S1). The Dianthus platform was used as an initial single-concentration biophysical triage assay, as described previously [23].

### 2.4. Microscale Thermophoresis

Twelve-point serial dilutions of NGM1 or its commercially available analogs were prepared from 250 µM to the low-nanomolar range and mixed with fluorescently labeled full-length His-tagged NOS1AP. The final DMSO concentration was maintained at 2.5%. After equilibration for 30 min at room temperature, samples were transferred to standard MST capillaries and analyzed using a Monolith NT.115 instrument with medium-to-high infrared-laser power and 60–80% LED excitation in the red detection channel. Dissociation constants were calculated using MO Affinity Analysis software (NanoTemper Technologies). Data points showing aggregation-associated behavior at the highest ligand concentrations were excluded from curve fitting. MST measurements and affinity analysis were performed as described previously [23].

### 2.5. Computational Pocket Analysis and Docking

The AlphaFold model of full-length human NOS1AP (UniProt O75052; 506 residues; AF-O75052-F1-v6) was prepared with PDBFixer 1.12. Missing heavy atoms were added, followed by hydrogen placement at pH 7.4. Candidate cavities were detected de novo with fpocket 3.0, which clusters Voronoi-derived alpha spheres and scores pockets using geometric and physicochemical properties. The highest-ranking site was selected for exploratory docking.

A three-dimensional conformer of NGM1 was generated in RDKit using the ETKDG algorithm and minimized with the MMFF94 force field. Meeko 0.7.1 was used to assign Gasteiger charges, define rotatable bonds, and prepare the rigid receptor in PDBQT format. Docking was performed with AutoDock Vina 1.2.5 using a 22.0 × 26.5 × 22.0 Å search box centered on the fpocket cavity [31], exhaustiveness of 12, and eight output poses. The highest-scoring pose was retained, and contacts were defined as receptor heavy atoms within 4.0 Å of a ligand heavy atom. The pose was treated as hypothesis-generating and was not used to infer quantitative affinity across the analog series.

### 2.6. Live-Cell NanoBRET Assay

Full-length human NOS1 bearing an N-terminal NanoLuc tag and full-length human NOS1AP bearing a C-terminal Venus tag were co-transfected into CHO-K1 cells. Cells were transferred to white 96-well plates and maintained until approximately 80% confluent. Before treatment, growth medium was removed, cells were washed twice with PBS, and HBSS was added. Cells were exposed to NGM1 or DMSO for 3 h. After NanoLuc substrate addition, donor emission at 460 nm and acceptor emission at 535 nm were measured with a Tecan plate reader. The NanoBRET ratio was calculated as acceptor emission divided by donor emission and normalized to the vehicle control.

### 2.7. GBM and Nonmalignant Cell Culture

Human U87 (from ATCC) and U251 (from Cytion) GBM cells and normal human astrocytes (from Cell Applications, INC) were maintained at 37 °C in a humidified atmosphere containing 5% CO_2_. U87 and U251 cells were cultured in Dulbecco’s modified Eagle medium supplemented with 10% fetal bovine serum and 1% penicillin–streptomycin. Normal human astrocytes were maintained in the supplier-recommended astrocyte growth medium. Cell identity and mycoplasma-negative status were confirmed before use.

### 2.8. Cell-Viability and Selectivity Studies

U87 cells, U251 cells, and normal human astrocytes were seeded into opaque 96-well plates at 3000, 4000, and 5000 cells per well, respectively, in a final volume of 100 µL per well, to maintain logarithmic growth throughout the experiment. After 24 h, cells were treated with vehicle or **NGM1** at concentrations ranging from 1 to 150 µM. The final DMSO concentration was identical across all conditions (0.1% *v*/*v*). After 72 h, viability was measured using CellTiter-Glo^®^ Luminescent Cell Viability Assay (from Promega), and signals were normalized to vehicle-treated cells. Luminescence was recorded using a Tecan plate reader, and signals were normalized to vehicle-treated cells.

Concentration–response curves were fitted using four-parameter nonlinear regression. IC_50_ values were calculated from three biologically independent experiments, each containing three technical replicates per concentration. The selectivity index was defined as the normal-astrocyte IC_50_ divided by the corresponding GBM-cell IC_50_. When 50% inhibition was not reached at the highest concentration tested, the IC_50_ and selectivity index were reported as lower-bound estimates.

### 2.9. Quantification of p53 and CDK6 by ELISA

U87 cells (2.0 × 10^5^ cells per well in 6-well plates) were treated with vehicle or **NGM1** at 5, 10, 20, or 30 µM for 24 h. Cells were collected in a lysis buffer (RIPA buffer, Thermo Fisher Scientific, 89,900) containing protease inhibitors, and total protein concentrations were determined using Pierce BCA Protein Assay Kit, Thermo Fisher Scientific, 23,225. Total p53 and CDK6 concentrations were measured using Human p53 SimpleStep ELISA Kit, Abcam, ab171571 and Human CDK6 SimpleStep ELISA Kit, Abcam, ab289900 according to the manufacturers’ instructions. Analyte concentrations were normalized to total lysate protein and then expressed relative to the corresponding vehicle control. Three biologically independent experiments were performed.

### 2.10. EdU-Incorporation Assay

DNA synthesis was evaluated using Click-iT EdU Alexa Fluor 488 Imaging Kit, Thermo Fisher Scientific, C10337. U87 cells (1.0 × 10^4^ cells per well in black, clear-bottom 96-well plates) were treated with vehicle or **NGM1** at 5, 10, 20, or 30 µM for 24 h and then incubated with EdU according to the assay manufacturer’s instructions. Cells were fixed, permeabilized, and labeled using click chemistry. Nuclei were counterstained with DAPI, and the percentage of EdU-positive nuclei was determined from multiple fields using Leica DMi8 automated fluorescence microscope. At least three fields were analyzed per well, and three biologically independent experiments were performed.

### 2.11. Cell-Cycle Analysis

U87 cells (2.0 × 10^5^ cells per well in 6-well plates) were exposed to vehicle or **NGM1** at 10, 20, or 30 µM for 24 h. Cells were harvested, washed with PBS, fixed in ice-cold 70% ethanol, and stored at 4 °C before analysis. Fixed cells were washed, treated with RNase A, and stained with propidium iodide. DNA content was measured by flow cytometry, and the percentages of cells in G_0_/G_1_, S, and G_2_/M phases were estimated using cell-cycle modeling software. Debris and doublets were excluded using prespecified gating criteria. Three biologically independent experiments were performed. Seeding densities were optimized for each cell type based on their respective growth and attachment characteristics to maintain suitable cell density and avoid excessive confluence during the treatment period.

### 2.12. Annexin V/Propidium–Iodide Analysis

Apoptotic cell death was evaluated after 48 h of exposure to vehicle or **NGM1** at 10, 20, or 30 µM. Cells were collected together with their corresponding culture supernatants, washed with PBS, and stained with eBioscience Annexin V Apoptosis Detection Kit FITC, Thermo Fisher Scientific, 88-8005-72. Flow-cytometric analysis classified cells as viable, early apoptotic, late apoptotic, or necrotic. Compensation settings and gating thresholds were established using unstained and single-stained controls. Three biologically independent experiments were performed.

### 2.13. Quantitative Reverse-Transcription PCR

U87 cells (2.0 × 10^5^ cells per well in 6-well plates) were treated with vehicle or **NGM1** at 5, 10, 20, or 30 µM for 24 h. Total RNA was isolated, treated with DNase, and reverse-transcribed into cDNA. Quantitative PCR was performed using validated primers for TP53, CDKN1A, and CDK6. GAPDH was used as the reference transcript after confirming its stability under the experimental conditions. RT-qPCR was performed using the following primer pairs (5′ → 3′): TP53, forward GAGGTTGGCTCTGACTGTACC and reverse TCCGTCCCAGTAGATTACCAC; CDKN1A, forward TGTCCGTCAGAACCCATGC and reverse AAAGTCGAAGTTCCATCGCTC; CDK6, forward TGGAGACCTTCGAGCACC and reverse CACTCCAGGCTCTGGAACTT; and GAPDH, forward ACAACTTTGGTATCGTGGAAGG and reverse GCCATCACGCCACAGTTTC. Primer specificity and amplification efficiency were verified before analysis. GAPDH was used as the reference transcript after confirming its stability under the experimental conditions. Relative transcript abundance was calculated using the 2^−ΔΔCt^ method and normalized to vehicle-treated cells. Three biologically independent experiments were performed.

### 2.14. Statistical Analysis

Values are presented as mean ± SD from three biologically independent experiments unless otherwise stated. Concentration–response curves were fitted using four-parameter nonlinear regression, and IC_50_ values are reported as mean ± SD of the three independently fitted values. ELISA, EdU-incorporation, and qPCR datasets were analyzed by one-way ANOVA followed by Dunnett’s test. Cell-cycle and Annexin V/propidium–iodide data were evaluated using one-way ANOVA with multiplicity-adjusted comparisons against vehicle. Statistical significance was defined as *p* < 0.05.

## 3. Results

### 3.1. AS-MS Identifies a New NOS1AP-Binding Chemotype

**Figure 1:**
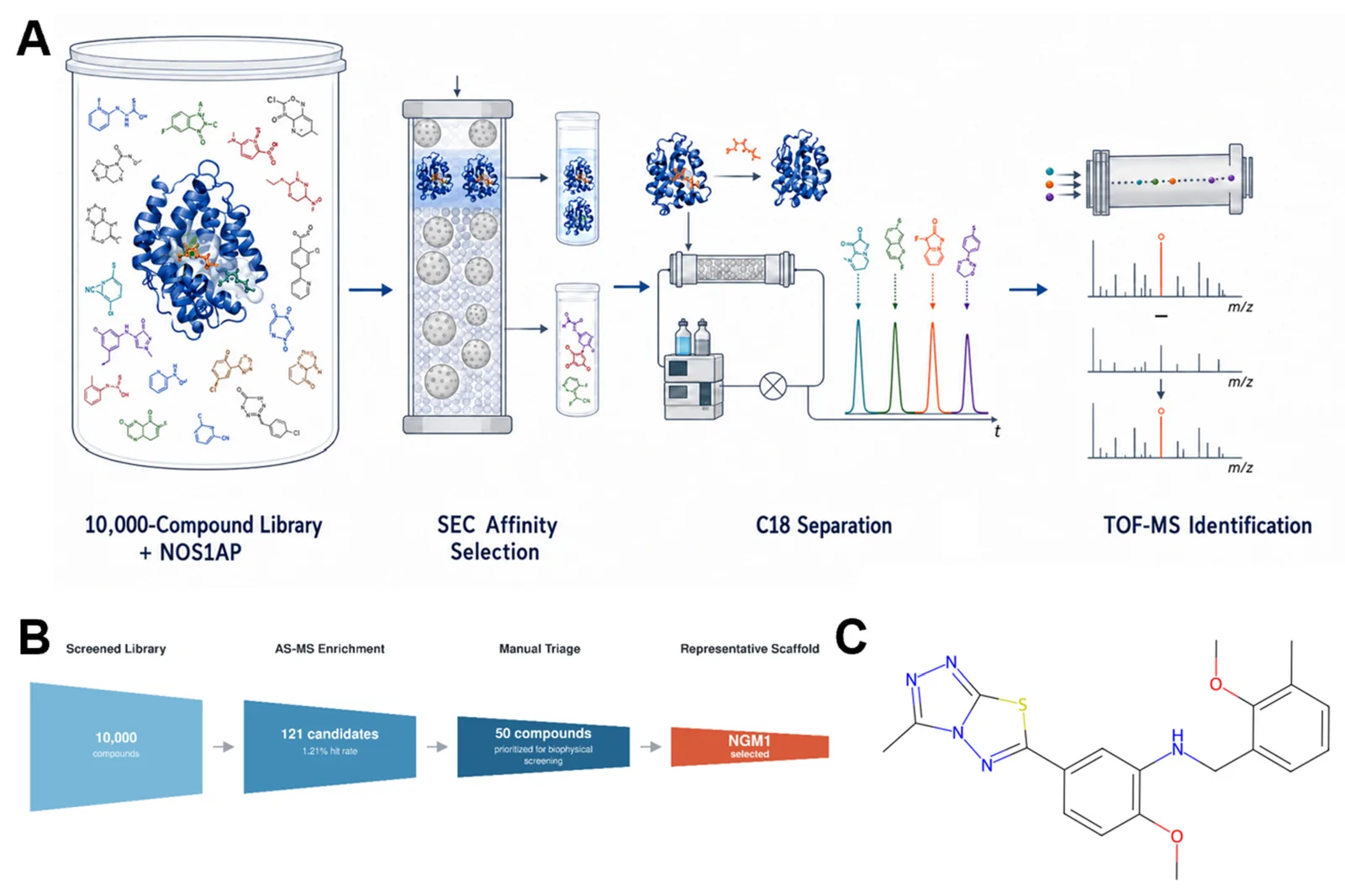
**Discovery workflow and identification of NGM1 as an NOS1AP-binding candidate.** (**A**) AS-MS workflow. (**B**) Enrichment and triage of the primary candidates. (**C**) Chemical structure of **NGM1**.

To identify chemical starting points for NOS1AP engagement, an AS-MS campaign was conducted against a 10,000-compound diversity library (Figure 1A). This label-free approach enables direct detection of protein–ligand complexes in solution without relying on a predefined catalytic site, a knowledge of the true binding pocket, or an existing ligand template, a necessity for an adaptor protein such as NOS1AP, which lacks the structural features that typically anchor conventional screening campaigns. Following incubation of recombinant NOS1AP with pooled compound mixtures and separation of bound from unbound species, mass spectrometric analysis identified 121 protein-associated candidates relative to protein-free controls, corresponding to an apparent primary enrichment rate of 1.21% (Figure 1B).

Manual inspection of these primary hits removed chemically reactive, unstable, or structurally redundant candidates, narrowing the set to 50 compounds that were advanced to single-concentration biophysical triage (Figure 1B). Among these, **NGM1** (Figure 1C) produced the strongest and most reproducible response and was selected as the representative scaffold for subsequent concentration-dependent characterization. Collectively, this campaign reduced a chemically diverse starting library to a focused, experimentally validated set of NOS1AP-binding candidates, establishing an initial chemical entry point for an adaptor protein previously considered outside the scope of conventional small molecule targeting strategies.

### 3.2. NGM1 Directly Engages NOS1AP in Solution

**Figure 2:**
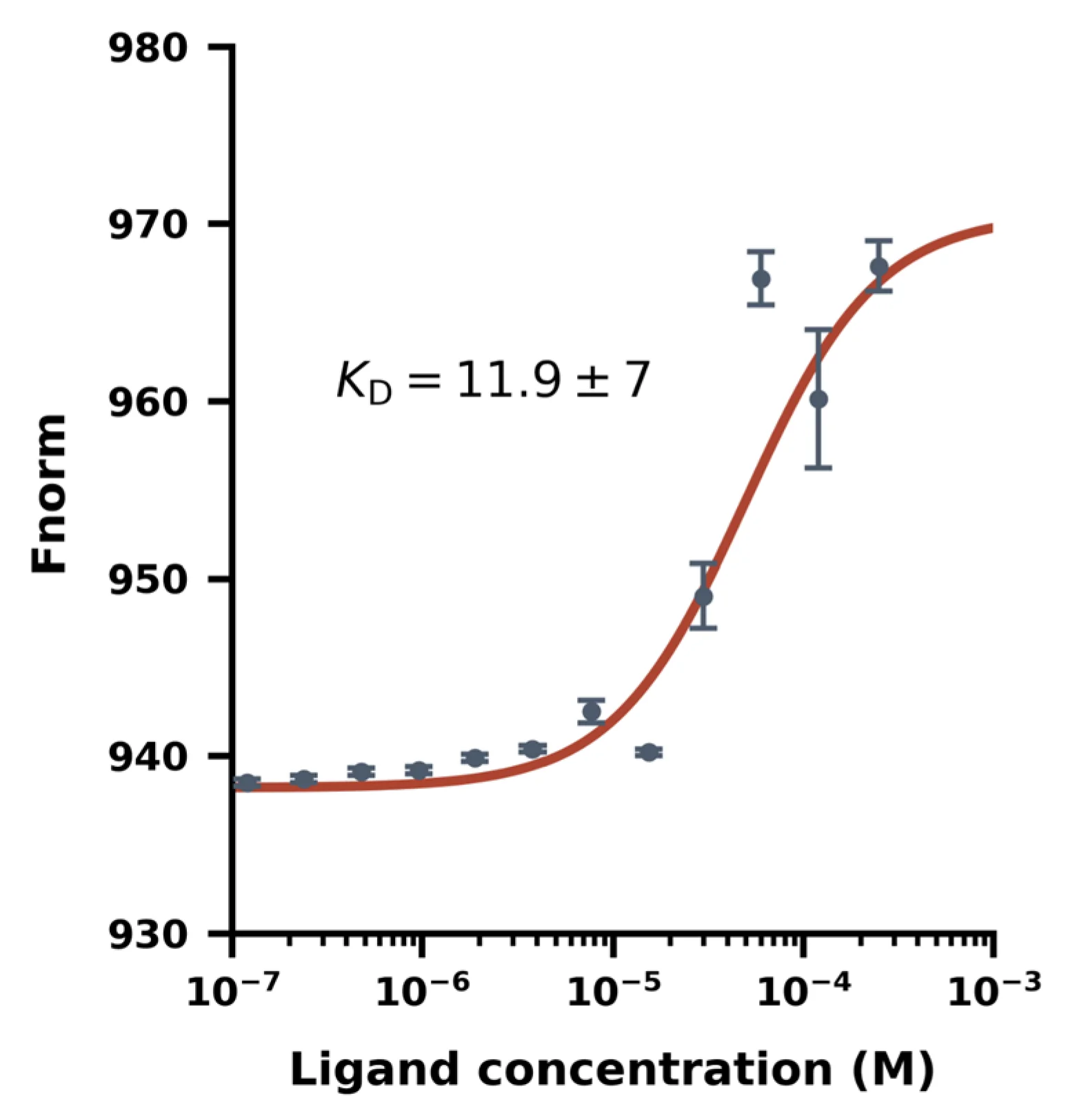
MST concentration-response curve for the binding of **NGM1** to NOS1AP. (*n* = 3, Error bars display SEM).

Microscale thermophoresis (MST) analysis was performed to orthogonally validate the direct interaction between **NGM1** and recombinant NOS1AP identified in the AS-MS screen. Titration of **NGM1** against fluorescently labeled NOS1AP produced a clear, concentration-dependent change in thermophoretic mobility, consistent with a specific binding interaction rather than a nonspecific or artifactual response. Nonlinear regression of the resulting binding curve yielded an apparent dissociation constant (Kd) of 11.9 ± 7 µM (Figure 2).

The resulting Kd is consistent with the affinity range typically observed for early, screening-derived fragment-like scaffolds prior to medicinal chemistry optimization, and is broadly comparable to hit-to-lead starting points reported for other adaptor-protein and protein–protein interaction targets. Taken together, these biophysical data support a direct, concentration-dependent association between **NGM1** and NOS1AP, while underscoring the need for an additional, orthogonal binding method, such as surface plasmon resonance (SPR) or isothermal titration calorimetry (ITC), to refine the affinity estimate during subsequent lead optimization.

### 3.3. Focused Analog Testing Defines Preliminary Recognition Features

**Figure 3:**
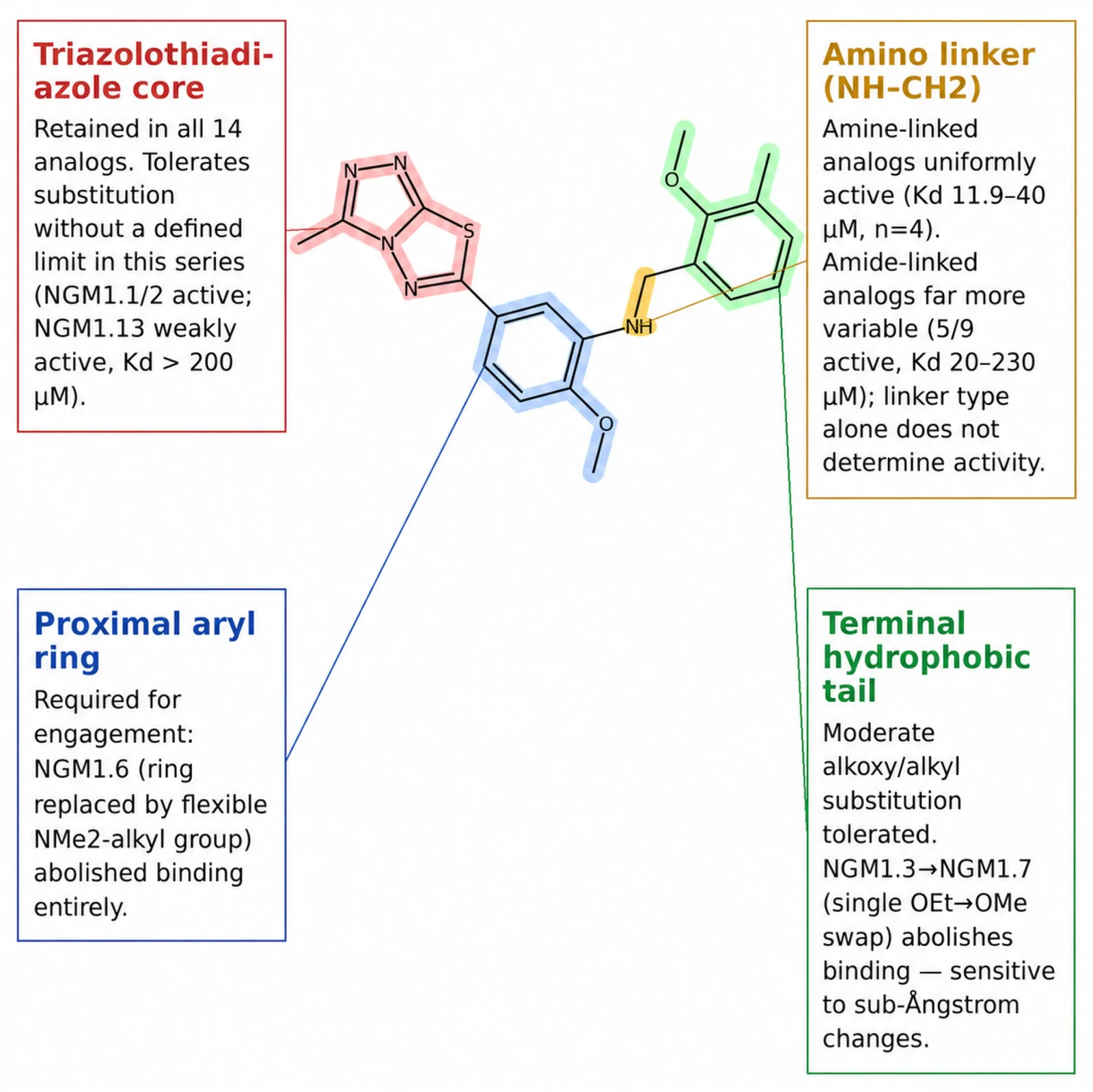
**Structure-Activity Relationship Summary for the NGM1 Chemotype.** The **NGM1** scaffold is shown with its four structural regions colored and annotated with the empirical conclusions drawn from the MST Kd data in Table 1: the triazolothiadiazole core (red), the proximal aryl ring (blue), the amino linker (yellow), and the terminal hydrophobic tail (green). Individual compound structures and Kd values for all fourteen analogs are provided in Table 1.

To assess the chemical tractability of the **NGM1** scaffold, a focused SAR-by-catalog approach was undertaken using the same fourteen compounds (**NGM1** and analogs **NGM1.1**–**NGM1.13**) and MST-derived Kd values described above (Table 1). A directly interpretable matched pair within this set is **NGM1.3** and **NGM1.7**, which are identical in every respect except for a single terminal alkyl group on the benzamide ring (para-ethoxy in **NGM1.3** versus para-methoxy in **NGM1.7**). This single-atom change was sufficient to convert a measurable interaction (**NGM1.3**, Kd = 50 μM) into no detectable binding (**NGM1.7**). Because this pair differs at only one position, the difference in binding suggests that NOS1AP-L recognition by this chemotype may be sensitive to changes in the terminal substituent; however, additional matched-pair analogs will be required to confirm this relationship (Figure 3).

Grouping compounds by linker chemistry revealed a second trend (Figure 3). The four compounds bearing a secondary amine linkage to the distal ring, either a benzylic NH-CH2 linker (**NGM1**, **NGM1.9**) or a direct/ether-tethered biaryl connection lacking a linker nitrogen entirely (**NGM1.1**, **NGM1.2**), were active, with Kd values of 11.9–40 μM (mean 23.9 μM). In contrast, the nine compounds bearing an amide linkage were far more variable: only five of nine (**NGM1.3**, **NGM1.4**, **NGM1.8**, **NGM1.11**, **NGM1.13**) showed detectable binding, with weaker affinities on average among the active subset (Kd 20–230 μM, mean 77.2 μM), and the remaining four (**NGM1.5**, **NGM1.7**, **NGM1.10**, **NGM1.12**) showed no detectable binding at all. This suggests that, while an amide-linked terminal ring is occasionally tolerated (e.g., **NGM1.8**, Kd = 20 μM), the amine-linked chemotype engages CAPON more consistently and with less sensitivity to the exact terminal substitution pattern. Complete removal of the proximal aromatic ring (**NGM1.6**, which replaces it with a flexible dialkylaminomethyl group) abolished binding entirely, indicating this ring, not merely the adjacent heteroatom, is potentially implicated in engagement (Figure 3).

No clean trend was observed for the identity of the core alkyl substituent (methyl versus ethyl) on the triazolothiadiazole ring considered in isolation: within the amide-linked subset, both core variants produced both active and inactive compounds. Bulky or symmetric ortho, ortho-disubstitution on the terminal ring (**NGM1.5**, **NGM1.13**) and fused bicyclic terminal rings (**NGM1.10**) were generally associated with reduced or abolished binding, though this trend has at least one clear exception (**NGM1.8**, whose symmetric 3,4,5-triethoxy substitution retains activity at Kd = 20 μM), suggesting substitution geometry and symmetry, rather than steric bulk alone, may govern tolerance at this position.

It is important to note that the current SAR analysis is based on a limited set of commercially available analogs, most of which incorporate multiple simultaneous structural modifications; the **NGM1.3**/**NGM1.7** pair is a notable exception and the only true single-point comparison in this set. As a result, most of the trends described above should be interpreted as associations rather than isolated causal effects. Future studies involving systematic, matched-pair analog design, building on the **NGM1.3**/**NGM1.7** result, will be required to more precisely define structure–activity relationships for this chemotype.

### 3.4. Structural Modeling Proposes a Ligandable Site on NOS1AP

**Figure 4:**
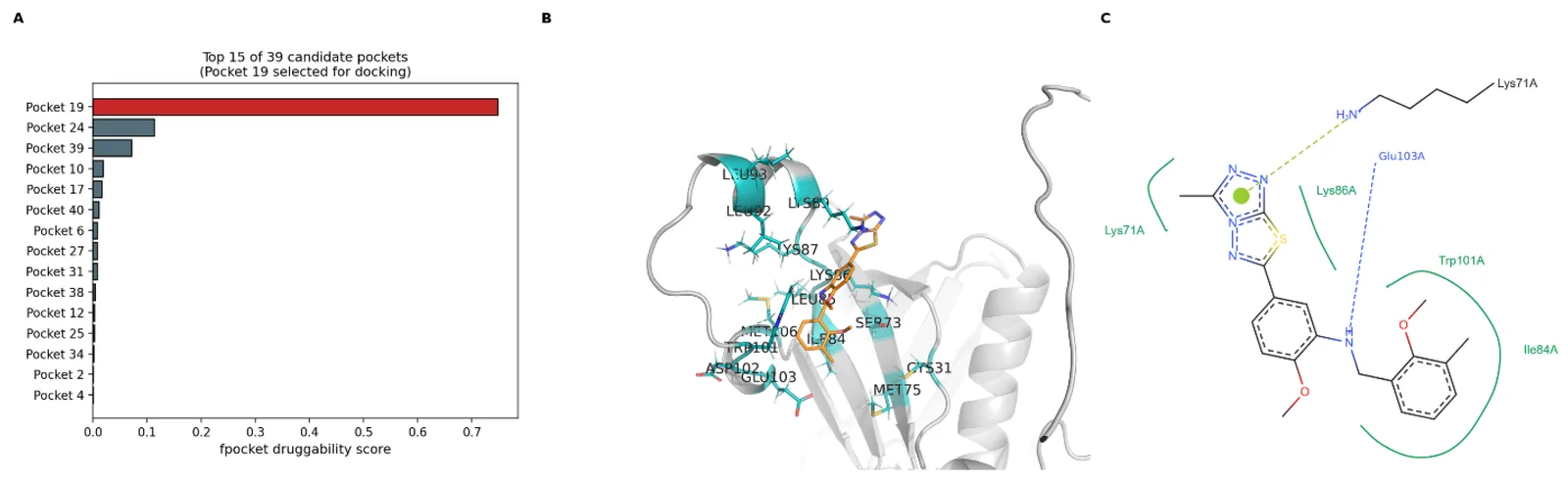
**Pocket Detection and Docking of NGM1 on the AlphaFold CAPON Model.** (**A**) fpocket druggability scores for the top 15 of 39 pockets identified de novo on the AlphaFold-predicted CAPON structure; the selected pocket (red) scores far above all others. (**B**) AlphaFold CAPON structure (cartoon, gray) with the top-ranked AutoDock Vina pose of **NGM1** (orange sticks) in the identified pocket; contacting residues (blue/teal sticks) include Lys71, Ile84, Leu85, Lys86–Lys88, Trp101, Glu103, and Met106. (**C**) Schematic two-dimensional interaction diagram of the same pose, highlighting a representative subset of contacts: Lys71 (cation-π and hydrophobic), Lys86 and Ile84 (hydrophobic), Trp101 (hydrophobic/aromatic), and Glu103 (hydrogen bond with the linker NH). The full set of nine contact residues identified by heavy-atom proximity (<4.0 Å) is given in panel (**B**) and the main text.

Given the absence of a crystal structure for NOS1AP (CAPON), we obtained a structural model by using the AlphaFold-predicted structure of full-length CAPON. To identify candidate binding sites, we ran fpocket, an open-source geometry- and machine-learning-based pocket detection algorithm, directly on this structure. Of 39 pockets identified, one showed a druggability score (0.748) far exceeding all others (≤0.113 for every remaining pocket; Figure 4A), and was lined by Cys31, Ser73, Met75, Ile84, Leu85, Lys86, Lys87, Lys89, Leu92, Leu93, Trp101, Asp102, Glu103, and Met106.

The parent compound **NGM1** was docked into this site using AutoDock Vina. The top-ranked pose (predicted affinity −6.43 kcal/mol) placed **NGM1** in contact (within 4.0 Å) with Lys71, Ile84, Leu85, Lys86, Lys87, Lys88, Trp101, Glu103, and Met106 (Figure 4B), overlapping closely with the pocket residues identified by fpocket (Figure 4A). Because docking scoring functions such as Vina are not expected to reliably rank closely related congeneric analogs, docking was not extended to the **NGM1**.**1**–**NGM1.13** analog series; the structure–activity trends among these analogs are instead derived directly from their MST-determined Kd values, independently of this docking result. Contacts in this pose fall into three groups (Figure 4C): a high proportion of lysine residues (Lys71, Lys86, Lys87, Lys88) forming electrostatic/polar contacts, one acidic residue (Glu103) capable of a polar or salt-bridge interaction, one aromatic residue (Trp101) positioned for a possible π-contact with the ligand’s heterocyclic core, and three hydrophobic residues (Leu85, Ile84, Met106) contacting the terminal aryl tail.

### 3.5. NGM1 Modulates the NOS1AP–NOS1 Complex in Living Cells

**Figure 5:**
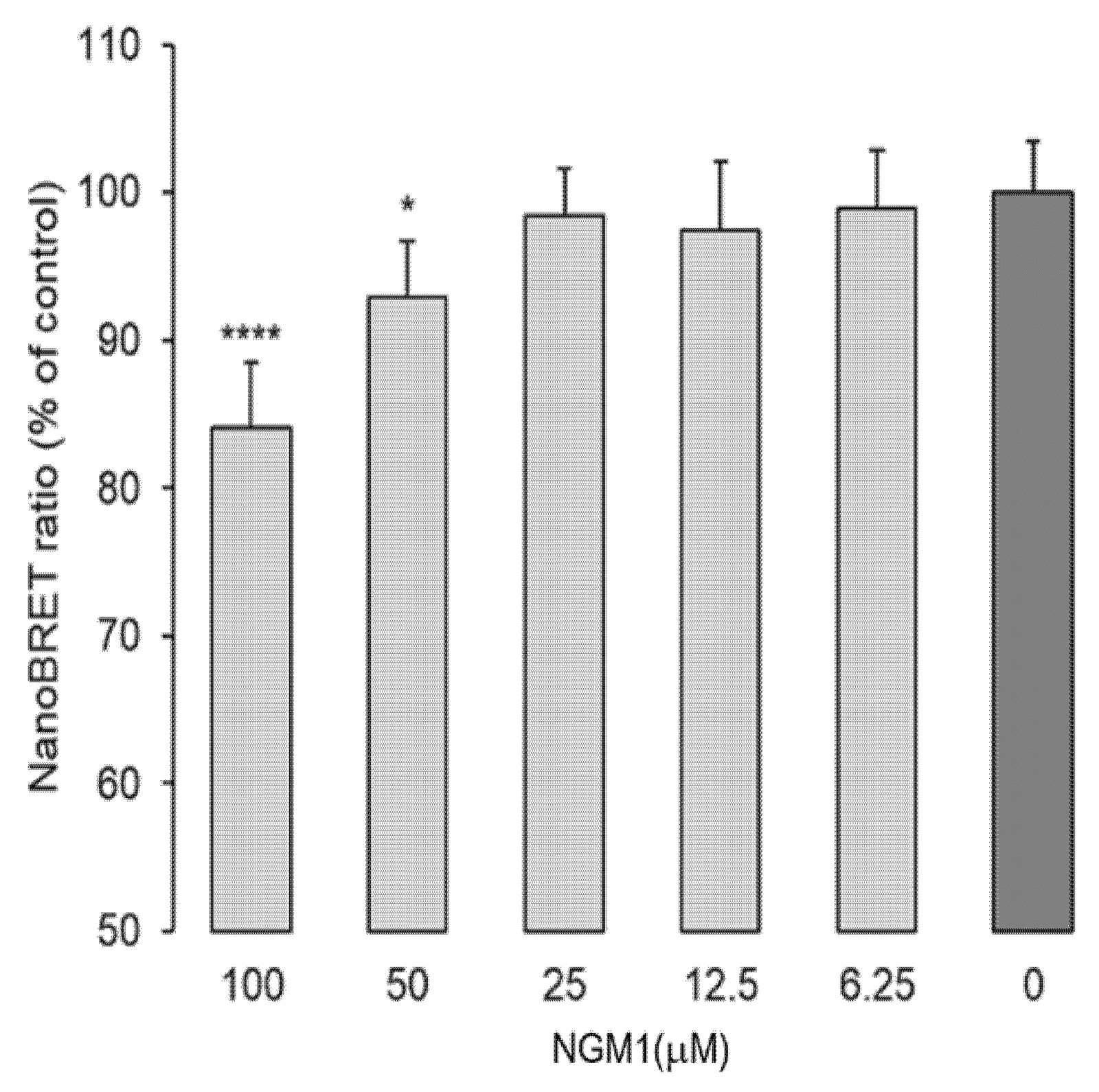
**Compound NGM1 produces a concentration-dependent reduction in the NOS1-NOS1AP NanoBRET signal.** CHO-K1 cells co-expressing NanoLuciferase-tagged NOS1 (N-terminal) and Venus-tagged CAPON (C-terminal) were treated with indicated concentrations of Compound **NGM1**. NanoBRET ratio was measured and expressed as a percentage of the vehicle (0.1% DMSO). Data are presented as mean ± SEM (*n* = 5). Statistical significance was assessed by one-way ANOVA [F(5, 24) = 10.75, *p* = 1.6 × 10^−5^] followed by Dunnett’s multiple comparisons test against the vehicle (0.1% DMSO). Adjusted *p* values: 100 µM, *p* = 1.5 × 10^−6^; 50 µM, *p* = 0.046; 25 µM, *p* = 0.12; 12.5 µM, *p* = 0.057; 6.25 µM, *p* = 0.34. * *p* < 0.05, **** *p* < 0.0001 vs. vehicle control.

The cellular activity of **NGM1** was assessed using NanoBRET between tagged NOS1AP and NOS1 in CHO-K1 cells. Compound exposure produced a concentration-dependent reduction in the normalized BRET signal. At 100 µM, the signal was approximately 80% of vehicle, and a statistically significant reduction was also observed at 50 µM (Figure 5). The incomplete response is consistent with partial modulation by an early low-micromolar scaffold. Because BRET reports proximity rather than direct intracellular binding, these results indicate perturbation of the reporter complex but do not independently define whether the compound occupies the modeled pocket or fully dissociates endogenous NOS1AP–NOS1 complexes.

### 3.6. NGM1 Reduces Viability in Two GBM Models

**Figure 6:**
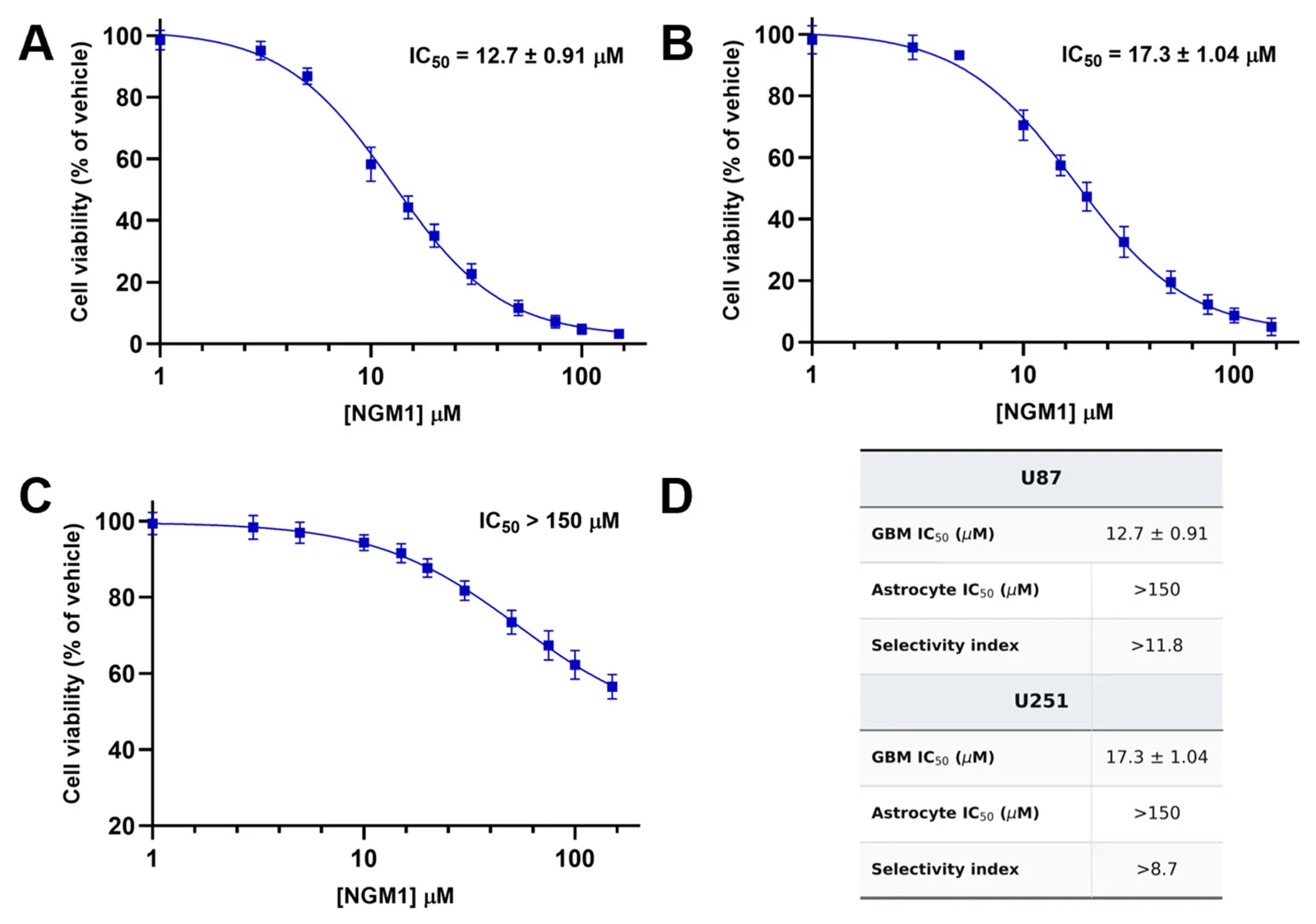
**Antiproliferative activity and cellular selectivity of NGM1.** (**A**) Concentration–response analysis of U87-cell viability after 72 h of **NGM1** exposure. (**B**) Concentration–response analysis of U251-cell viability. (**C**) Viability of normal human astrocytes over the same concentration range. (**D**) Comparison of the IC_50_ values and selectivity indices. Because astrocyte viability remained above 50% at 150 µM, the astrocyte IC_50_ and corresponding selectivity indices are presented as lower-bound estimates. Symbols represent mean viability and error bars represent SD from three biologically independent experiments, each containing three technical replicates. Curves represent four-parameter nonlinear-regression fits.

To determine whether **NGM1** engagement translated into an antiproliferative cellular response, its effects on viability were evaluated in two independent GBM cell models (U87 and U251 cells). **NGM1** produced a concentration-dependent reduction in viability in both GBM models after 72 h, with IC_50_ values of 12.7 ± 0.91 µM in U87 cells and 17.3 ± 1.04 µM in U251 cells (Figure 6A,B).

Normal human astrocytes were substantially less sensitive to **NGM1**. Astrocyte viability remained above 50% at the highest concentration tested, resulting in an IC_50_ estimate of >150 µM (Figure 6C). These results corresponded to selectivity indices of >11.8 relative to U87 cells and >8.7 relative to U251 cells (Figure 6D). The findings indicate preferential activity for **NGM1** in GBM cells compared with nonmalignant astrocytes under the conditions examined.

### 3.7. NGM1 Produces Molecular and Cellular Changes Consistent with p53/CDK6-Associated Cell-Cycle Arrest

**Figure 7:**
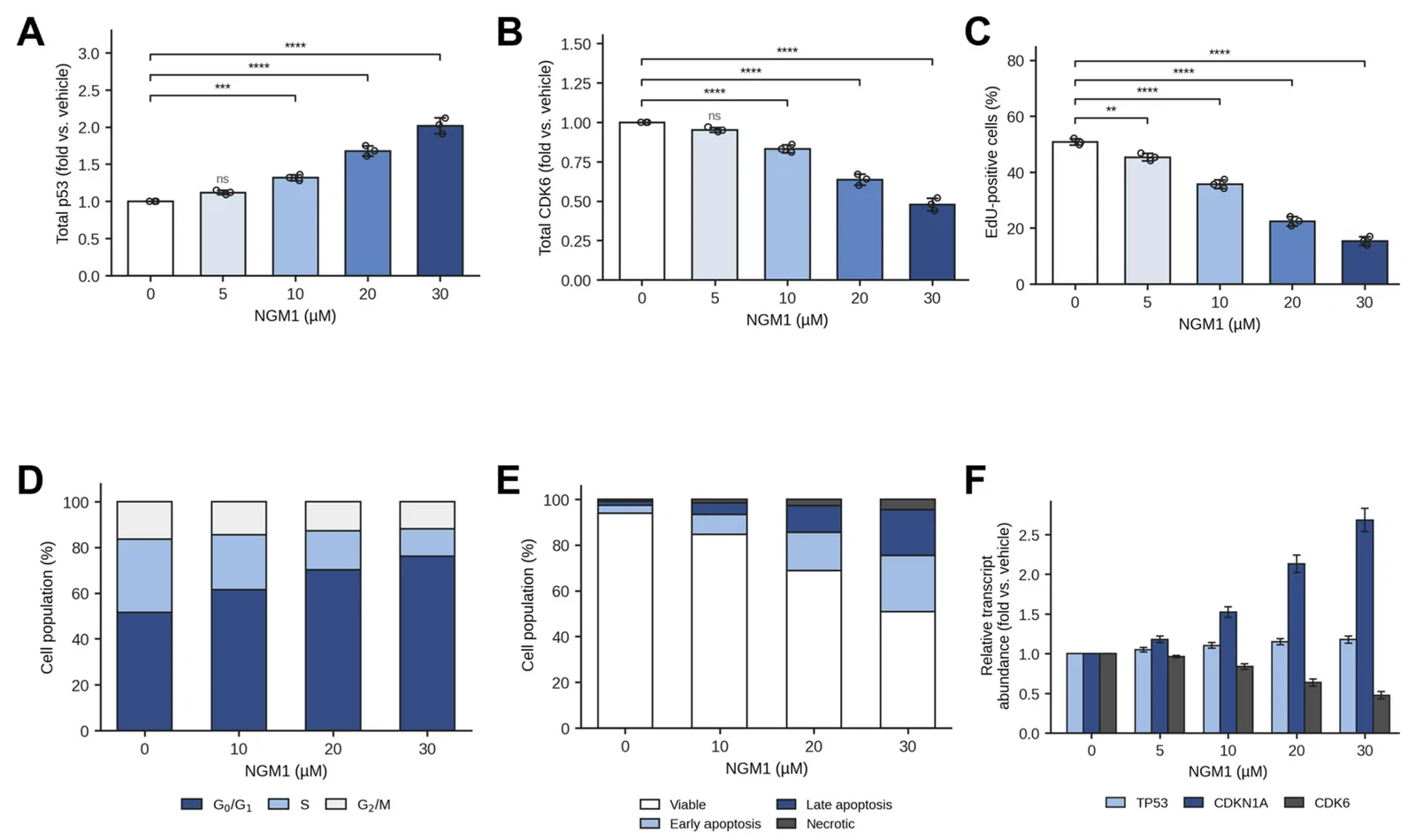
**Evaluation of molecular and cellular responses to NGM1.** (**A**) Total p53 abundance measured by quantitative ELISA in U87-cell lysates after 24 h of NGM1 treatment. (**B**) Total CDK6 abundance measured in the same lysates. ELISA values were normalized to total lysate protein and expressed relative to vehicle (0.1% DMSO). (**C**) Percentage of EdU-positive U87 cells after 24 h of treatment. (**D**) Distribution of cells across G_0_/G_1_, S, and G_2_/M phases determined by propidium-iodide flow cytometry after 24 h. (**E**) Annexin V/propidium-iodide classification of viable, early-apoptotic, late-apoptotic, and necrotic populations after 48 h. (**F**) Relative TP53, CDKN1A, and CDK6 transcript abundance measured by RT-qPCR after 24 h. In (**A**–**C**), points represent individual biologically independent experiments and bars denote mean ± SD from *n* = 3 independent experiments; comparisons vs. vehicle were performed by one-way ANOVA with Dunnett’s post hoc test ** *p* < 0.01, *** *p* < 0.001, **** *p* < 0.0001, ns *p* > 0.05. In (**D**,**E**), stacked bars show mean percentages from *n* = 3 independent experiments; each population/class was analyzed separately by one-way ANOVA with Dunnett’s post hoc test vs. vehicle.

To investigate the cellular processes accompanying **NGM1**-mediated growth inhibition, total p53 and CDK6 abundance was quantified by ELISA after 24 h of treatment in U87 cells. **NGM1** produced concentration-dependent changes in both proteins. At 30 µM, total p53 abundance increased to 2.02 ± 0.11-fold relative to vehicle, whereas CDK6 abundance decreased to 0.48 ± 0.04-fold (Figure 7A,B). The molecular changes were accompanied by reduced DNA synthesis. The proportion of EdU-positive cells declined from 50.8 ± 1.2% in vehicle-treated cultures to 35.7 ± 1.6%, 22.5 ± 1.7%, and 15.4 ± 1.6% following treatment with 10, 20, and 30 µM **NGM1**, respectively (Figure 7C).

Cell-cycle analysis demonstrated a corresponding accumulation in G_0_/G_1_ phase. The G_0_/G_1_ population increased from 51.5 ± 0.7% under vehicle conditions to 61.5 ± 0.6%, 70.1 ± 1.0%, and 76.1 ± 1.1% following treatment with 10, 20, and 30 µM **NGM1**, respectively. This change was accompanied by a reduction in the S-phase population from 32.1 ± 0.5% to 12.0 ± 0.6% at 30 µM (Figure 7D). Annexin V/propidium-iodide analysis showed that the reduction in proliferation preceded a later increase in apoptotic cell death. After 48 h, the combined early- and late-apoptotic population increased from approximately 5% under vehicle conditions to approximately 14%, 29%, and 45% following exposure to 10, 20, and 30 µM **NGM1**, respectively (Figure 7E). Necrosis remained comparatively limited, reaching approximately 5% at 30 µM.

RT-qPCR analysis provided additional support for a cell-cycle-associated response. At 30 µM, CDKN1A expression increased to 2.68 ± 0.15-fold, whereas CDK6 expression decreased to 0.48 ± 0.05-fold relative to the vehicle. TP53 transcript abundance changed only modestly, reaching 1.18 ± 0.05-fold (Figure 7F). The divergence between the pronounced increase in p53 protein and the comparatively limited change in TP53 mRNA is compatible with post-transcriptional regulation of p53 abundance. Together, these findings are consistent with p53/CDK6-associated G_0_/G_1_ arrest, reduced DNA synthesis, and subsequent apoptotic cell death. Genetic depletion or rescue experiments will be required to establish NOS1AP dependence.

## 4. Discussion

The present study provides an initial pharmacological framework for investigating NOS1AP in GBM. A ligand-discovery campaign that did not depend on catalytic activity or prior structural knowledge yielded a small molecule chemotype capable of associating with recombinant NOS1AP, altering a live-cell NOS1AP–NOS1 proximity readout, and suppressing growth in two GBM cell models. Together, these results move NOS1AP from genetic and pathway-level observation toward experimental chemical tractability.

Several features strengthen this conclusion. The primary AS-MS enrichment was followed by orthogonal concentration-dependent biophysical analysis rather than being interpreted as binding on its own. The analog set showed that small chemical changes could abolish the MST response, which is more consistent with structure-sensitive recognition than with uniform nonspecific association. Moreover, the live-cell NanoBRET experiment provided evidence that the scaffold can perturb a NOS1AP-containing complex in a cellular environment.

In parallel with the molecular binding and NanoBRET observations, NGM1 produced measurable cellular effects in the two GBM models examined. **NGM1** reduced viability in two independently evaluated GBM models. Importantly, normal human astrocytes remained above 50% viability at the highest concentration tested, producing lower-bound selectivity indices of >11.8 for U87 cells and >8.7 for U251 cells. These results suggest that the cellular response is not explained solely by concentration-dependent general cytotoxicity, although broader evaluation in additional nonmalignant neural cell types will be needed to define the therapeutic window.

The mechanistic studies revealed a coherent sequence of cellular changes. Increased p53 protein abundance and reduced CDK6 abundance were accompanied by induction of CDKN1A, accumulation of cells in the G_0_/G_1_ phase, and suppression of EdU incorporation. Apoptosis became more apparent at the later 48 h time point, whereas necrosis remained limited. This temporal pattern is consistent with an initial antiproliferative response followed by apoptotic cell death. The modest change in TP53 transcript abundance relative to the increase in p53 protein further suggests that **NGM1** may influence p53 stability or turnover rather than strongly increasing TP53 transcription.

The difference between the low-micromolar biochemical affinity of **NGM1** and the higher concentrations required to modulate the NanoBRET reporter also warrants consideration. This difference may reflect cellular permeability, intracellular free-compound concentration, reporter geometry, protein abundance, or incomplete disruption of a multiprotein complex. Accordingly, **NGM1** should be regarded as an initial chemical probe and optimization scaffold rather than a fully validated therapeutic lead or in vivo therapeutic candidate. Future medicinal-chemistry and delivery studies should seek to improve affinity, cellular potency, selectivity, metabolic properties, and central nervous system exposure. The predicted pocket and docking pose are particularly provisional because they rely on an AlphaFold model and have not been challenged by mutagenesis. These structural results are most useful for designing testable substitutions and pocket mutants rather than asserting a definitive binding mode.

An important limitation of the present study is that the cellular effects of NGM1 cannot yet be attributed exclusively to NOS1AP engagement. Although NGM1 binds recombinant NOS1AP-L and alters the NOS1AP–NOS1 NanoBRET signal, these observations do not exclude contributions from NOS1AP-independent or off-target mechanisms to the observed effects on cell viability, cell-cycle progression, and associated signaling pathways. Establishing target dependency will require genetic approaches, such as NOS1AP depletion and rescue experiments, as well as complementary target-engagement studies. Accordingly, NGM1 should presently be considered an initial NOS1AP-binding chemical probe rather than a fully validated NOS1AP-selective pharmacological agent.

A further limitation is that the cellular activity of NGM1 was evaluated in only two established GBM cell lines. Although activity was observed in both U87 and U251 cells, these models do not capture the molecular and phenotypic heterogeneity of GBM. Future studies should therefore evaluate NGM1 in additional molecularly diverse models, including patient-derived GBM cells, glioma stem-like cells, and three-dimensional tumor models.

Future studies should first establish whether the antiproliferative activity of NGM1 is NOS1AP-dependent through NOS1AP depletion, rescue, and binding-deficient mutant experiments. Orthogonal biophysical and cellular target-engagement assays will be required to confirm direct intracellular engagement and refine the apparent affinity estimate. In parallel, systematic matched-pair analog design should define the structure-activity relationship and improve potency, selectivity, and physicochemical properties. Evaluation in patient-derived GBM cells, glioma stem-like models, and three-dimensional tumor systems should precede pharmacokinetic, brain-exposure, tolerability, and efficacy studies in orthotopic GBM models.

In summary, **NGM1** provides a starting chemical probe for NOS1AP and reveals a potential route to pharmacologically interrogate this adaptor in GBM. The findings justify systematic analog design and mechanistic studies, but they do not yet establish an in vivo therapeutic candidate. This distinction is important for positioning NOS1AP-directed chemistry as an emerging opportunity while maintaining an appropriately evidence-based interpretation.

## 5. Conclusions

A combined AS-MS, biophysical, computational, live-cell interaction, and GBM phenotyping workflow identified a small-molecule modulator of NOS1AP. **NGM1** directly associated with NOS1AP, altered NOS1AP-NOS1 proximity, and inhibited viability in two GBM cell lines with comparatively lower activity in nonmalignant astrocytes. Potency optimization, additional target validation, and brain-directed delivery studies are required to determine whether this chemical starting point can progress toward a GBM therapeutic strategy.

## Figures and Tables

**Table 1:** **Chemical structures and NOS1AP binding affinities of compound NGM1 derivatives.** Kd values were determined by microscale thermophoresis (MST).

Compound	MST Kd (µM)	Chemical Structure
**NGM1**	11.9	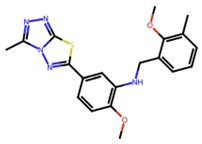
**NGM1.1**	20.8	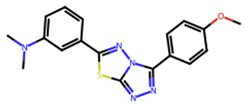
**NGM1.2**	40	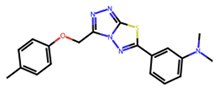
**NGM1.3**	50	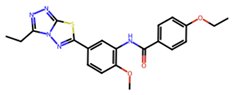
**NGM1.4**	48	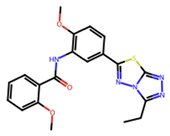
**NGM1.5**	N.D.	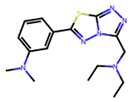
**NGM1.6**	N.D.	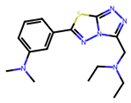
**NGM1.7**	N.D.	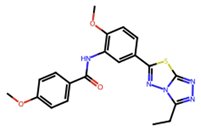
**NGM1.8**	20	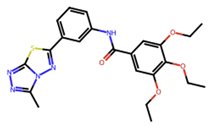
**NGM1.9**	23	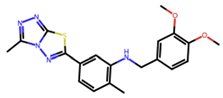
**NGM1.10**	N.D.	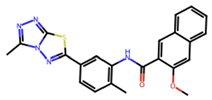
**NGM1.11**	38	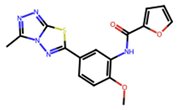
**NGM1.12**	N.D.	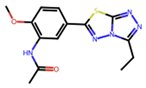
**NGM1.13**	230	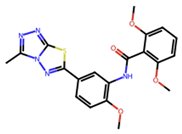

N.D., no detectable binding under the tested conditions.

## Data Availability

The data supporting the findings of this study are available from the corresponding author upon reasonable request.

## Supplementary Materials

The following supporting information can be downloaded at https://www.mdpi.com/article/10.3390/life16091519/s1: Figure S1: Single-concentration Dianthus screening of AS-MS-derived candidate compounds. Fifty compounds selected from the initial AS-MS screen were evaluated by Dianthus at a final compound concentration of 250 µM in the presence of 2.5% DMSO. The reference consisted of PBST buffer containing 2.5% DMSO. Red points indicate compounds with signals outside the threshold defined as the mean reference signal ± 3 SD, whereas black points indicate compounds within the threshold range. Dotted lines indicate the upper and lower threshold values.
