# Supplementary material for: Small Molecule Modulation of NOS1AP for Molecularly Targeted Glioblastoma Therapy
Source: Life (Basel). 2026 Sep 11;16(9):1519. doi: 10.3390/life16091519 (PMC13608063; doi:10.3390/life16091519)
Supplement: Supplementary file 1 [file life-16-01519-s001.zip › life-4492563-supplementary.pdf]

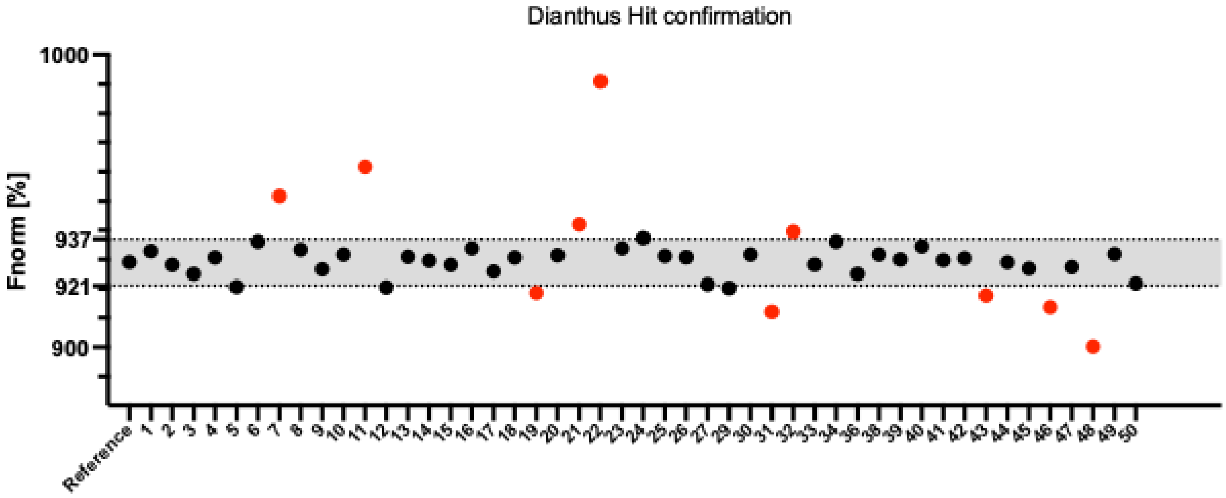

Figure S1. Single-concentration Dianthus screening of AS-MS-derived candidate compounds. Fifty compounds selected from the initial AS-MS screen were evaluated by Dianthus at a final compound concentration of 250 µM in the presence of 2.5% DMSO. The reference consisted of PBST buffer containing 2.5% DMSO. Red points indicate compounds with signals outside the threshold defined as the mean reference signal ± 3 SD, whereas black points indicate compounds within the threshold range. Dotted lines indicate the upper and lower threshold values.
